# Clinicopathological and prognostic significance of circRNAs in lung cancer: A systematic review and meta-analysis

**DOI:** 10.1097/MD.0000000000025415

**Published:** 2021-04-09

**Authors:** Yuxuan Zheng, Jie Hu, Yishuai Li, Ran Hao, Yixin Qi

**Affiliations:** a School of Nursing, Hebei Medical University, Shijiazhuang, Hebei; b Department of Respiratory Medicine, First Hospital of Jilin University, Changchun, Jilin, China; c Department of Pharmacology and Toxicology, School of Medicine, University of Louisville, Louisville, KY; d Morning Star Academic Cooperation, Shanghai; e Department of Science and Technology, Hebei Medical University; f Department of Thoracic Surgery, Hebei Provincial Chest Hospital; g Department of Breast Center, The Fourth Hospital of Hebei Medical University, Shijiazhuang, Hebei, China.

**Keywords:** biological mechanism, circular RNA, clinicopathological characteristics, lung cancer, prognosis, systematic review

## Abstract

**Background:**

Circular RNAs (circRNAs) regulate multiple pathways during lung cancer pathogenesis. Apart from functional significance, many circRNAs have been shown to be associated with clinicopathological characteristics and predict lung cancer prognosis. Our aim is to summarize the expanding knowledge of clinical roles of circRNAs in lung cancer.

**Methods:**

A thorough search of literature was conducted to identify articles about the correlation between circRNA expression and its prognostic and clinicopathological values. Biological mechanisms were summarized.

**Results:**

This study included 35 original articles and 32 circRNAs with prognostic roles for lung cancer. Increased expression of 25 circRNAs and decreased expression of 7 circRNAs predicted poor prognosis. For non-small cell lung cancer, changes of circRNAs were correlated with tumor size, lymph node metastasis, distant metastasis, tumor node metastasis (TNM) stage, and differentiation, indicating the major function of circRNAs is to promote lung cancer invasion and migration. Particularly, meta-analysis of ciRS-7, hsa_circ_0020123, hsa_circ_0067934 showed increase of the 3 circRNAs was associated with positive lymph node metastasis. Increase of ciRS-7 and hsa_circ_0067934 was also related with advanced TNM stage. The biological effects depend on the general function of circRNA as microRNA sponge.

**Conclusions:**

CircRNAs have the potential to function as prognostic markers and are associated with lung cancer progression and metastasis.

## 1 Introduction

Lung cancer is the leading cause of cancer-related deaths all over the world.^[1,2]^ One out of every 4 cancer deaths is due to lung cancer.^[1]^ In China, lung cancer has also become an enormous socioeconomic and public health threat. Chinese patients account for more than one-third of all newly diagnosed cases every year.^[2]^ Among all cancers, lung cancer ranks first for men and second for women in China, and the incidence for women is still increasing.^[2,3]^ Pathologically, lung cancer has been recognized as a heterogeneous disease.^[4]^ Traditional classification is based on histology and immunohistochemical biomarkers. Over 85% of the cases belong to non-small cell lung cancer (NSCLC), which can be further subclassified mainly into adenocarcinoma (LUAD, ∼50%), squamous cell carcinoma (LUSC, ∼40%), large cell carcinoma, and some neuroendocrine tumors (∼10%).^[5]^ The majority of the remaining 15% is highly aggressive and fatal small-cell lung cancer.^[5]^ Understanding of lung cancer at tissue level has not yielded satisfying curable treatments as the 5-year survival has barely improved during last few decades with a dismal rate varying from 4% to 17% based on stage and region.^[6]^ In China, the average overall survival (OS) of advanced NSCLC is only 13.7 months.^[7]^ However, technological developments have allowed us to understand lung cancer to the deeper genetic and molecular levels.^[8,9]^ Current theory of pathogenesis of lung cancer is a multifactorial and integrated paradigm involving genetic and epigenetic alterations, individual immune status and tumor microenviroment.^[4,10,11]^ Moreover, many researches have been translated into clinical trials focusing on targeting specific tyrosine kinase receptors, immune checkpoints, tumor-mesenchymal cells interaction, and angiogenesis.^[12–14]^ Although current milestone progress is made predominantly in the field of genes and their protein products, epigenetic regulators, such as noncoding RNAs (ncRNAs), are burgeoning, and more and more studies are showing ncRNAs not only play crucial roles in pathogenesis, treatment responsiveness, and drug resistance, but also have the potential to function as a biomarker for diagnosis and prognosis of lung cancer.^[15,16]^

The last decade has witnessed an unexpected and fascinating discovery of diverse ncRNAs with distinguished regulatory roles. NcRNAs are generally divided into small linear ncRNAs (<200 nucleotides), long linear ncRNAs (>200 nucleotides), and circular RNAs (circRNAs).^[17,18]^ Unlike linear ncRNAs, the 3′ and 5′ ends of circRNA are covalently jointed together in a process called backsplicing, which is an alternative splicing of pre-mRNA.^[19,20]^ Characteristics of circRNAs include high stability and abundance, developmental and cell type specificity, and highly evolutionary conservation across species.^[21]^ The biological functions of circRNAs have not been completely elucidated. One general function of circRNAs is acting as microRNA (miRNA) sponges.^[22]^ Given that miRNA is well-known to inhibit mRNA translation, circRNA is able to increase gene expression by competing with mRNA for miRNA.^[23]^ Another aspect is that circRNAs can bind to RNA-associated proteins, which is directly involved in gene transcription.^[24]^ The roles of circRNAs are being explored extensively in human diseases, such as ischemic heart disease, diabetes, and Alzheimer disease.^[25]^ For example, ciRS-7, one of the most studied circRNAs, has been found to increase insulin secretion from pancreatic β islet cells by binding to and inhibiting the function of miRNA-7 as its super sponge.^[26]^ Moreover, cirRNAs are also associated with several hallmarks of cancers, including sustaining proliferative signaling, evading growth suppressors, activating invasion and metastasis, inducing angiogenesis, and evading cell death and senescence.^[27]^ Via the same sponge mechanism, ciRS-7 has been shown to promote oncogene epidermal growth factor receptor (EGFR) expression and inhibit tumor suppressor gene *KLF4* expression, therefore, inducing tumor initiation and progression.^[28]^ CircRNAs are also proposed as diagnostic biomarkers of cancer in a meta-analysis.^[29]^

Particularly in lung cancer, many studies have been conducted to compare expression levels of a specific circRNA between cancerous and adjacent noncancerous tissues, and to evaluate its clinical significance as a diagnostic or prognostic marker.^[30]^ On the other aspect, mechanisms of different circRNAs in lung cancer are being revealed.^[31]^ As people are gaining insights into how circRNAs regulate vital steps in lung cancer development, circRNAs are showing promise to become new drug targets. It is the fast-growing amount of circRNA research in lung cancer and the great clinical translational potential that make summarizing current data on circRNAs in lung cancer urgent and necessary. Our aim in this study is to perform a systemic review and meta-analysis of the biological function and clinicopathological significance of circRNAs with prognostic value in lung cancer. Although several linear ncRNAs have also been shown to regulate multiple biological processes and associate with diagnosis and prognosis of lung cancer,^[32,33]^ we focuses only on circRNAs because currently established mechanism of circRNAs in lung cancer is mediated as the sponge of miRNA, the characteristic of which is better explored and understood than that of long ncRNA.^[34,35]^ Furthermore, the number of studies exploring either clinicopathological or prognostic significance of linear ncRNA in lung cancer is limited for a systematic review and meta-analysis compared to circRNA.^[36]^ To our surprise, based on our thorough database search, all the studies meeting our criteria were conducted in China, which makes our study limited to specific Chinese genetic background.

## 2 Materials and methods

### 2.1 Identification of relevant studies

PubMed, Embase, Web of Science were searched to identify literature on the topic of prognostic significance of circRNA expression in patients with lung cancer. The database surveys were conducted on March 4, 2020. The keywords used were as follows: “lung,” “pulmonary,” “neoplasms,” “neoplasia,” “cancer,” “tumor,” “carcinoma,” “malignancy,” “malignant neoplasm,” “circRNA,” “circular RNA,” “circ.”

### 2.2 Criteria of filtering studies

The inclusion criteria included 2 items:

(1) All the patients in the study underwent biopsy and the diagnosis of lung cancer was confirmed by experienced pathologists;
(2) The correlation between circRNA expression and OS was reported in the form of Kaplan–Meier survival curve or hazard ratio.

The exclusion criteria included 4 items:

(1) Abstracts, letters, case reports, reviews, summary of conference, editorials, commentaries, and nonclinical studies were filtered out.
(2) Studies that were not written in English were not included.
(3) Original articles focusing exclusively on biological function of circRNA in cell lines.
(4) CircRNA expression was measured in the peripheral blood instead of lung tissue.

### 2.3 Data extraction

Two investigators independently extracted data and a third investigator got involved if there was a discrepancy. A consensus was reached after discussion among the 3 investigators. The following data were extracted from an original study: fist author, journal name, journal impact factor, circRNA name, number of patients included, circRNA expression level, circRNA high expression percentage, cut-off standard, type of survival indicator, expression level predicting poor prognosis, follow-up time, clinicopathological factor, biological effects, and mechanism. Clinicopathological characteristics reviewed in this study included age, sex, smoking status, histopathological classification, differentiation, tumor size, lymph node metastasis, distant metastasis, and tumor node metastasis (TNM) stage. This study was approved by the Ethics and Research Committee of Fourth Hospital of Hebei Medical University.

### 2.4 Statistical analysis

STATA 12.0 was used to pool odds ratios (ORs) and corresponding 95% confidence intervals (CIs) for assessing the strength of the association between expression of a specific circRNA and relevant clinicopathological characteristics. If the combined OR >1 and its 95% CI does not include 1, this clinicopathological feature was regarded to be significantly related to change of this circRNA expression. *Q* test and *I*^2^ test were performed to estimate the heterogeneity between various studies. If *P* > .05 and *I*^2^ < 50%, we considered there was no heterogeneity and the fixed effects model was used to calculate the pooled OR. Otherwise, the random effects model was used.^[37,38]^

## 3 Results

### 3.1 Screening and characteristics of studies with prognosis-predictive circRNAs in lung cancer

**Figure 1:**
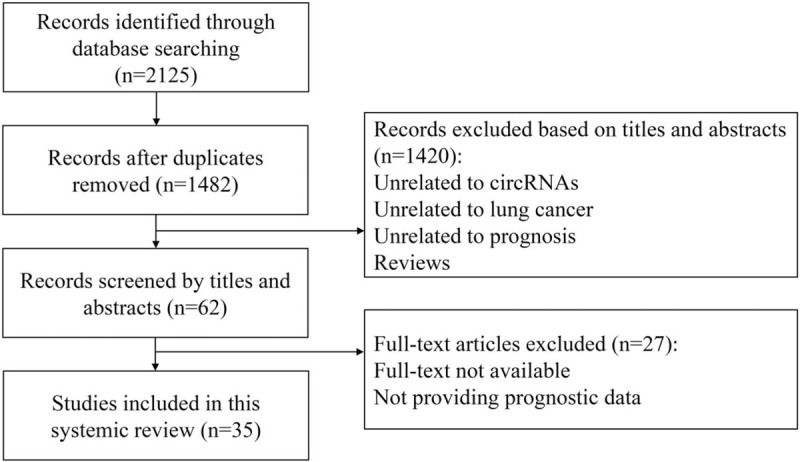
Flowchart of identifying relevant studies.

After the initial search of Pubmed, Embase, and Web of Science, we identified 2125 candidate papers. Due to duplication, 643 papers were removed. Then, titles and abstracts were scanned, and 1420 papers were excluded because they were either review articles or unrelated to circRNA, lung cancer, or prognosis. Next, full-text articles were assessed, and 27 papers were excluded for not providing prognostic data. Based on the above steps, 35 papers were included for this systemic review (Fig. 1).

Basic characteristics, including first author, journal, impact factor, circRNA name, number of patients, and circRNA expression level, were listed in Table 1. All the studies were conducted in China during the last 2 years, indicating exploration of prognostic significance of circRNAs has been popular, at least in part of the world. Number of patients varied from 35 to 159 (median, 71). Jiali Xu et al examined 2 circRNAs, hsa_circ_103827 and hsa_circ_000122, in lung cancer in their paper while other authors examined only 1 circRNA. Because of that, we had 36 studies in those 35 papers. On the other hand, 3 studies focused on ciRS-7, 2 studies focused on hsa_circ_0067934 and 2 studies focused on hsa_circ_0020123. The expression levels of 25 circRNAs increased while the remaining 7 decreased. Quantitative real-time polymerase chain reaction was applied to measure circRNA expression level in lung tissue in all the studies except that conducted by Mantang Qiu, where RNA chromogenic in situ hybridization in tissue microarray was used. Twenty-three studies pointed out specifically that they collected samples in surgery from patients without previous chemotherapy or radiotherapy.

**Table 1:** Summary of basic characteristics of include studies on circRNAs with prognostic values in lung cancer.

First author, yr	Journal	Impact factor	CircRNA	Number of patients	Expression
Jingquan Han, 2018	Biochem Biophys Res Commun.	2.705	circ-BANP	59	Increased
Baiquan Qiu,^‡^ 2019	J Cell Physiol.	4.522	circFGFR3	63	Increased
Mantang Qiu,^∗,‡^ 2018	Cancer Res.	8.378	circPRKCI	89	Increased
Yuan Wang,^†^ 2019	Gene.	2.638	circ-PRMT5	90	Increased
Si Qin, 2019	Biomed Pharmacother.	3.743	circPVT1	90	Increased
Xiaofei Zhang, 2018	Onco Targets Ther.	3.046	ciRS-7	60	Increased
Chongyu Su, 2018	J Cell Mol Med.	4.658	ciRS-7	128	Increased
B. YAN, 2018	Eur Rev Med Pharmacol Sci.	2.721	ciRS-7	132	Increased
Yuanshan Yao, 2019	Biomed Pharmacother.	3.743	has_circ_0001946	72	Increased
Jingchun An,^†^ 2019	Biochem Biophys Res Commun.	2.705	has_circ_0003645	59	Increased
Wanjun Yu,^‡^ 2018	Onco Targets Ther.	3.046	hsa_circ_0003998	60	Increased
You Zhou,^†^ 2019	Biochem Biophys Res Commun.	2.705	hsa_circ_0004015	35	Increased
Yi Qi,^†^ 2018	Gene.	2.638	hsa_circ_0007534	98	Increased
Xiuying Li, 2019	Eur Rev Med Pharmacol Sci.	2.721	hsa_circ_000984	155	Increased
Lingchi Ding, 2018	Oncol Lett.	1.871	hsa_circ_001569	56	Increased
Yongsheng Li,^†^ 2018	Biochem Biophys Res Commun.	2.705	hsa_circ_0016760	83	Increased
Danhua Qu, 2018	Am J Cancer Res.	4.737	hsa_circ_0020123	80	Increased
Jingru Wan, 2019	Biochem Biophys Res Commun.	2.705	hsa_circ_0020123	55	Increased
Xiwang Ying,^‡^ 2019	Mol Genet Genomic Med.	2.448	hsa_circ_0020732	78	Increased
Chengjun Liu,^†^ 2019	Onco Targets Ther.	3.046	hsa_circ_0023404	36	Increased
Guohua Liu, 2019	Biochem Biophys Res Commun.	2.705	hsa_circ_0025033	80	Increased
Qinguang Zou,^‡^ 2018	Oncol Lett.	1.871	hsa_circ_0067934	79	Increased
J. Wang, 2018	Eur Rev Med Pharmacol Sci.	2.721	hsa_circ_0067934	159	Increased
Wei Han,^†^ 2019	Biochem Biophys Res Commun.	2.705	hsa_circ_0087862	40	Increased
Fucheng Zhao, 2018	Biosci Rep.	2.535	hsa_circ_100833	43	Increased
Juntao Yao, 2017	Pathol Res Pract.	1.794	hsa_circ_100876	101	Increased
Liang Zong, 2018	Biomed Pharmacother.	3.743	hsa_circ_102231	57	Increased
Wei Liu, 2018	Biochem Biophys Res Commun.	2.705	hsa_circ_103809	44	Increased
Jiali Xu, 2018	Am J Transl Res.	3.266	hsa_circ_103827	40	Increased
Jiali Xu, 2018	Am J Transl Res.	3.266	hsa_circ_000122	40	Decreased
Tongmiao Liu,^‡^ 2018	Biochem Biophys Res Commun.	2.705	hsa_circ_0001649	53	Decreased
Lin Wang, 2019	Cancer Sci.	4.751	hsa_circ_0002346	92	Decreased
Yuanshan Yao, 2019	Biochem Biophys Res Commun.	2.705	hsa_circ_0006427	94	Decreased
Binbin Zhang, 2019	Cancer Biol Ther.	2.879	hsa_circ_0007874	63	Decreased
Liu Yang, 2018	Respir Res.	3.829	hsa_circ_0046264	99	Decreased
Daishi Chen, 2018	Cell Cycle.	3.259	hsa_circ_100395	69	Decreased

CircRNA expression level of lung tissue was measured in all the studies. Quantitative real-time PCR was used as the method except Mantang Qiu's study (labeled with ^∗^), in which RNA chromogenic in situ hybridization in tissue microarray was used. Seven authors (labeled with ^†^) did not mention how they acquired the samples or whether patient had undergone some certain treatment. Six authors (labeled with ^‡^) mentioned they collected the samples in surgery but did not mention if the patients had received other treatment. The remaining authors pointed out specifically that they acquired the samples in surgery and only from patients without chemotherapy or radiotherapy. circRNAs = Circular RNAs.

### 3.2 Association between circRNA expression level and OS in lung cancer patients

Table 2 summarized the study designs and results of various prospective cohorts exploring the relationship between change of circRNA expression in cancerous tissue and patients’ survival. Except 3 studies, the remaining 33 studies provided exact high circRNA expression percentage, ranging between 42% and 68% (median, 51%). This variation was dependent on the cut-off standard for dividing patients into high or low cirRNA expression group. Nineteen studies used median as cut-off value while another 11 studies used mean. However, 6 studies did not state their choice of cut-off standard. The shortest follow-up period was less than 20 months and the longest time was 150 months. Eighteen studies chose 60 months as 5-year survival is well accepted to monitor cancer mortality. Most of the studies, 34 out of 36, employed OS as the outcome. Each study either provided hazard ratio and 95% CI directly or presented Kaplan–Meier survival curve to establish the prognostic role of individual circRNA. High expressions of circ-BANP, circFGFR3, circPRKCI, circ-PRMT5, circPVT1, ciRS-7, hsa_circ_0003645, hsa_circ_0001946, hsa_circ_0003998, hsa_circ_0004015, hsa_circ_0007534, hsa_circ_000984, hsa_circ_001569, hsa_circ_0016760, hsa_circ_0020123, hsa_circ_0020732, hsa_circ_0023404, hsa_circ_0025033, hsa_circ_0067934, hsa_circ_0087862, hsa_circ_100833, hsa_circ_100876, hsa_circ_102231, hsa_circ_103809 and hsa_circ_103827 in lung cancer tissue were associated with poor prognosis, while low expressions of hsa_circ_000122, hsa_circ_0001649, hsa_circ_0002346, hsa_circ_0006427, hsa_circ_0007874, hsa_circ_0046264, and hsa_circ_100395 were associated with poor prognosis.

**Table 2:** Summary of prognostic significance of circRNAs in lung cancer.

CircRNA	CircRNA high expression percentage	Cut-off standard	Survival indicator	Expression level predicting poor prognosis	Follow-up (mo)
circ-BANP	47%	Not mentioned	Overall survival	High	60
circFGFR3	54%	Not mentioned	Overall survival	High	>80
circPRKCI	62%	Not mentioned	Overall survival	High	>80,<100
circ-PRMT5	50%	Median	Overall survival	High	>68,<85
circPVT1	48%	Median	Overall survival	High	60
ciRS-7	68%	Median	Overall survival	High	>80,<100
ciRS-7	60%	Mean	Overall survival	High	60
ciRS-7	50%	Median	Overall survival	High	>80,<100
hsa_circ_0003645	54%	Median	Overall survival	High	60
hsa_circ_0001946	53%	Mean	Overall survival	High	60
hsa_circ_0003998	Not specified	Not mentioned	Not specified	High	<40
hsa_circ_0004015	Not specified	Not mentioned	Overall survival	High	<60
hsa_circ_0007534	57%	Mean	Overall survival	High	60
hsa_circ_000984	52%	Median	Overall survival	High	60
hsa_circ_001569	52%	Mean	Overall survival	High	60
hsa_circ_0016760	54%	Mean	Overall survival	High	60
hsa_circ_0020123	50%	Median	Overall survival	High	>60
hsa_circ_0020123	51%	Mean	Overall survival	High	60
hsa_circ_0020732	50%	Median	Overall survival	High	>60, <80
hsa_circ_0023404	50%	Median	Overall survival	High	60
hsa_circ_0025033	55%	Mean	Overall survival	High	60
hsa_circ_0067934	52%	Median	Overall survival	High	80
hsa_circ_0067934	50%	Median	Overall survival	High	60
hsa_circ_0087862	50%	Mean	Overall survival	High	60
hsa_circ_100833	51%	Median	Overall survival	High	60
hsa_circ_100876	Not specified	Not mentioned	Overall survival	High	>40, <50
hsa_circ_102231	51%	Median	Not specified	High	60
hsa_circ_103809	50%	Median	Overall survival	High	80
hsa_circ_103827	50%	Median	Overall survival	High	>40, <50
hsa_circ_000122	50%	Median	Overall survival	Low	>40, <50
hsa_circ_0001649	42%	Mean	Overall survival	Low	60
hsa_circ_0002346	50%	Median	Overall survival	Low	>100, <120
hsa_circ_0006427	57%	Mean	Overall survival	Low	60
hsa_circ_0007874	49%	Median	Overall survival	Low	100
hsa_circ_0046264	56%	Median	Overall survival	Low	>16, <20
hsa_circ_100395	51%	Mean	Overall survival	Low	150

circRNAs = Circular RNAs.

### 3.3 Association between circRNA expression level and clinicopathological characteristics in lung cancer patients

Those circRNAs not only correlated with survival, but also associated with several clinicopathological features. Table 3 exhibited the relationship between change of circRNA expression and clinicopathological characteristics based on pathological classification of lung cancer. Most studies concentrated on NSCLC, regardless of subtypes. Tumor size, lymph node metastasis, distant metastasis, TNM stage, and differentiation were shown to relate to increase or decrease of different circRNAs. On the other hand, many circRNAs were associated with more than 1 factor. Furthermore, a small portion of the studies explored LUAD, a major subtype of NSCLC. For this specific subtype, tumor size, lymph node metastasis, and TNM stage were linked to circRNA level. Other factors were either found not correlated with circRNA level significantly or not explored by the authors. No study investigated this clinicopathological relationship in LUSC, and several studies did not clarify the pathological type of their lung cancerous tissue.

**Table 3:** Summary of clinicopathological significance of circRNAs with prognostic values in lung cancer.

		Change of circRNA expression	
Category	Clinicopathological factor	Increased	Decreased
NSCLC	Tumor size	circFGFR3	
		circ-PRMT5	
		circPVT1	
		ciRS-7^∗^	
		hsa_circ_0003998	
		hsa_circ_0004015	
	Lymph node metastasis	circFGFR3	hsa_circ_0001649
		circ-PRMT5	hsa_circ_0046264
		ciRS-7^†^	
		has_circ_0003645	
		hsa_circ_0003998	
		hsa_circ_0007534	
		hsa_circ_000984	
		hsa_circ_001569	
		hsa_circ_0016760	
		hsa_circ_0020123^‡^	
		hsa_circ_0025033	
		hsa_circ_0067934^§^	
		hsa_circ_0087862	
		hsa_circ_100833	
		hsa_circ_100876	
	Distant metastasis	hsa_circ_0067934^||^	
	TNM stage	circFGFR3	hsa_circ_0001649
		circ-PRMT5	hsa_circ_0046264
		circPVT1	
		ciRS-7^†^	
		has_circ_0003645	
		hsa_circ_0004015	
		hsa_circ_0007534	
		hsa_circ_000984	
		hsa_circ_001569	
		hsa_circ_0016760	
		hsa_circ_0020123^‡^	
		hsa_circ_0025033	
		hsa_circ_0067934^§^	
		hsa_circ_100833	
		hsa_circ_100876	
	Differentiation	circFGFR3	
		ciRS-7^¶^	
		hsa_circ_0004015	
		hsa_circ_001569	
		hsa_circ_0020123^#^	
		hsa_circ_0087862	
		hsa_circ_100833	
LUAD	Tumor size	circPRKCI	hsa_circ_0006427
		has_circ_0001946	
	Lymph node metastasis	hsa_circ_0020732	hsa_circ_0002346
		hsa_circ_102231	hsa_circ_0006427
	TNM stage	circPRKCI	hsa_circ_0002346
		has_circ_0001946	hsa_circ_0006427
		hsa_circ_0020732	
		hsa_circ_102231	
Not specified	Lymph node metastasis	circ-BANP	hsa_circ_100395
	TNM stage	circ-BANP	hsa_circ_100395

circRNAs = Circular RNAs, LUAD = adenocarcinoma, NSCLC = non-small cell lung cancer, TNM = tumor node metastasis.

∗ For the studies conducted by Chongyu Su and B. Yan.

† For all 3 studies concerning ciRS-7.

‡ For both studies concerning hsa_circ_0020123.

§ For both studies concerning hsa_circ_0067934.

|| For the study conducted by J. Wang.

¶ For the study conducted by Chongyu Su.

# For the study conducted by Danhua Qu.

Considering multiple studies were evaluating clinicopathological significance of ciRS-7, hsa_circ_0020123, and hsa_circ_0067934, we conducted meta-analysis for these 3 circRNAs. One of the 3 studies about ciRS-7 did not provide enough clinicopathological information. Only clinicopathological factors included in both studies for the above 3 cirRNAs were used for the analysis. As shown in Table 4, increased ciRS-7 was significantly associated with positive lymph node metastasis (pooled OR = 2.71, 95% CI: 1.40–5.26, *P* = .003, fixed effects) and advanced TNM stage (pooled OR = 3.06, 95% CI: 1.63–5.74, *P* = .001, fixed effects). However, there was no significant correlation between increased ciRS-7 and sex (OR = 0.71, 95% CI: 0.38–1.32, *P* = .279, fixed effects) or histopathological type (pooled OR = 1.04, 95% CI: 0.23–4.63, *P* = .956, random effects). One hundred eighty-eight patients were included in the meta-analysis for ciRS-7. Table 5 showed increase of hsa_circ_0020123 was associated with pathologically poorly differentiated tumors (pooled OR = 2.53, 95% CI: 1.24–5.16, *P* = .011, fixed effects) and positive lymph node metastasis (pooled OR = 3.36, 95% CI: 1.65–6.84, *P* = .001, fixed effects). Sex was not associated with risk of increase of hsa_circ_0020123 (pooled OR = 1.03, 95% CI: 0.52–2.04, *P* = .941, fixed effects). One hundred thirty-five patients were included for calculating combined OR. Similar to ciRS-7, Table 6 displayed that hsa_circ_0067934 elevation was also significantly associated with positive lymph node metastasis (pooled OR = 2.82, 95% CI: 1.62–4.92, *P* < .001, fixed effects) and advanced TNM stage (pooled OR = 2.91, 95% CI: 1.69–5.01, *P* < .001, fixed effects), and not related with sex (pooled OR = 1.32, 95% CI: 0.77–2.24, *P* = .314, fixed effects) or age (pooled OR = 1.34, 95% CI: 0.78–2.28, *P* = .288, fixed effects). Two hundred thirty-eight patients were included. This common clinicopathological significance shared by ciRS-7, hsa_circ_0020123, and hsa_circ_0067934 indicated change of expression levels of different circRNAs could serve as a universal predictor for tumor invasion and metastasis. More studies are needed to confirm our results and to explore the relationship between cirRNA level and other clinicopathological factors.

**Table 4:** Association of increased ciRS7 with clinicopathological characteristics.

					Heterogeneity		
Clinicopathological factor	Number of patients in group 1	Number of patients in group 2	OR (95% CI)	*P*-value	*I*^2^ (%)	*P*_*h*_-value	Model
Sex (male vs female)	116	72	0.71 (0.38, 1.32)	.279	<0.01	.323	Fixed
Histopathological type (LUAD vs LUSC)	82	106	1.04 (0.23, 4.63)	.956	78.5	.031	Random
Lymph node metastasis (positive vs negative)	73	115	2.71 (1.40, 5.26)	.003	<0.01	.504	Fixed
TNM stage (III + IV vs I + II)	92	96	3.06 (1.63, 5.74)	.001	14.7	.279	Fixed

Group 1 represents patients of male sex, LUAD subtype, positive lymph node metastasis and III or IV TNM stage, respectively. Group 2 represents female sex, LUSC subtype, negative lymph node metastasis, and I or II TNM stage, respectively. CI = confidence interval, LUAD = adenocarcinoma, LUSC = squamous cell carcinoma, OR = odds ratio, TNM = tumor node metastasis.

**Table 5:** Association of increased hsa_circ_0020123 with clinicopathological characteristics.

					Heterogeneity		
Clinicopathological factor	Number of patients in group 1	Number of patients in group 2	OR (95% CI)	*P*-value	*I*^2^ (%)	*P*_*h*_-value	Model
Sex (male vs female)	81	54	1.03 (0.52, 2.04)	.941	<0.01	.657	Fixed
Differentiation (poorly vs well/moderately)	57	78	2.53 (1.24, 5.16)	.011	<0.01	.491	Fixed
Lymph node metastasis (positive vs negative)	64	71	3.36 (1.65, 6.84)	.001	<0.01	.781	Fixed

Group 1 represents patients of male sex, poorly differentiated tumor and positive lymph node metastasis, respectively. Group 2 represents female sex, well/moderately differentiated tumor and negative lymph node metastasis, respectively. CI = confidence interval, OR = odds ratio.

**Table 6:** Association of increased hsa_circ_0067934 with clinicopathological characteristics.

					Heterogeneity		
Clinicopathological factor	Number of patients in group 1	Number of patients in group 2	OR (95% CI)	*P*-value	*I*^2^ (%)	*P*_*h*_-value	Model
Sex (male vs female)	151	87	1.32 (0.77, 2.24)	.314	<0.01	.690	fixed
Age (>60 vs <60)	104	134	1.34 (0.78, 2.28)	.288	<0.01	.951	fixed
Lymph node metastasis (positive vs. negative)	84	154	2.82 (1.62, 4.92)	<.001	<0.01	.740	fixed
TNM stage (III + IV vs I + II)	92	146	2.91 (1.69, 5.01)	<.001	<0.01	.707	fixed

Group 1 represents patients of male sex, >60 yr old, positive lymph node metastasis and III or IV TNM stage, respectively. Group 2 represents female sex, <60 yr old, negative lymph node metastasis and I or II TNM stage, respectively. CI = confidence interval, OR = odds ratio, TNM = tumor node metastasis.

## 4 Discussion

Our study systemically summarized current prognostic and clinicopathological roles of 32 circRNAs in patients with lung cancer, mostly NSCLC, throughout China. More than 2700 patients participated in at least 1 of the 36 studies. According to our inclusion criteria, changes of expression of all 32 circRNAs had been shown to be associated with either poor or good OS. Further clinicopathological characteristics correlation study also revealed that changes of majority of those circRNAs were predictive of positive lymph node metastasis and clinically advanced tumor stage, which indicated the functional roles of circRNAs in lung cancer could be affecting tumor invasion and progression.

Overall mechanisms of circRNAs are miRNA sponges in all the included studies. Although the exact role of certain circRNA is dependent on both its interactive miRNA and the function of this miRNA target in a specific biological pathway, most studies, 29 out of 36, exhibited increase of circRNA expression was predictive of bad clinical outcome. Among the 29 studies, 23 studies also included functional assays and confirmed the overall role of those circRNAs was promoting cancer. As shown in Table 7, for the 25 tumor-promoting circRNAs, 24 of them except hsa_circ_0020732 promoted proliferation on cellular level, and stimulated tumor growth if animal study was also conducted. Meanwhile, 10 circRNAs, including circ-BANP, circPVT1, ciRS-7, hsa_circ_0001946, hsa_circ_0007534, hsa_circ_000984, hsa_circ_0016760, hsa_circ_0020123, hsa_circ_0025033, and hsa_circ_0087862 were shown to inhibit apoptosis, further enhancing tumor viability. On the other hand, circ-BANP, cirFGFR3, circPRKCI, ciRS-7, hsa_circ_0003645, hsa_circ_0003998, hsa_circ_0004015, hsa_circ_0007534, hsa_circ_000984, hsa_circ_0016760, hsa_circ_0020123, hsa_circ_0020732, hsa_circ_0023404, hsa_circ_0025033, hsa_circ_0067934, hsa_circ_0087862, hsa_circ_100833, hsa_circ_102231, and hsa_circ_103809 could promote migration and/or invasion in vitro, corroborating clinical implication of advanced tumor stage and positive metastasis by increase of those circRNAs. Upregulation of circPRKCI and hsa_circ_0004015 conferred resistance to EGFR tyrosine kinase inhibitor gefitinib. Furthermore, related to both tumor progression and drug resistance, epithelial-mesenchymal transition (EMT) had been observed with high levels of hsa_circ_0007534, hsa_circ_000984, hsa_circ_0023404, and hsa_circ_0067934. Decrease of circRNA expression was less commonly seen, and low levels of 7 circRNAs were predictive of poor OS of lung cancer patients. The biological effects of 6 circRNAs out of 7 had been explored and they were categorized as tumor-suppressing circRNAs. Contrary to tumor-promoting circRNAs, increase of those circRNAs resulted in suppression of tumor proliferation, induction of apoptosis, inhibition of migration, invasion, and EMT.

**Table 7:** Summary of molecular mechanisms of circRNAs with prognostic values in lung cancer.

CircRNA	Overall role	Biological effects	Mechanism
circ-BANP	Promote tumor	In vitro: promote proliferation, migration and invasion, inhibit apoptosis; in vivo: promote propagation	Inhibition of miR-503 → upregulation of LARP1 → promote tumor
circFGFR3	Promote tumor	In vitro: promote proliferation and invasion	Inhibition of miR-22-3p → upregulation of Gal1, p-AKT, p-ERK1/2 → promote tumor
circPRKCI	Promote tumor	In vitro: promote proliferation and migration, enhance resistance to gefitinib; in vivo: promote growth	Inhibition of miR-545 and miR-589 → upregulation of E2F7 → downregulation of CDKN1A (P21) and upregulation of CCND1 (Cyclin D1) → promote tumor
circ-PRMT5	Promote tumor	In vitro: promote growth, decrease cells in G0/G1 phase, increase cells in S and G2/M phases; in vivo: promote growth	Inhibition of miR-377, miR-382 and miR-498 → upregulation of EZH2 → promote tumor
circPVT1	Promote tumor	In vitro and in vivo: promote proliferation, inhibit apoptosis	Inhibition of miR-497 → upregulation of Bcl-2 → promote tumor
ciRS-7	Promote tumor	In vitro: promote vitality and growth, inhibit apoptosis and G1/S arrest; in vivo: promote growth	Inhibition of miR-7 → upregulation of EGFR, CCNE1, PIK3CD → promote tumor
		in vitro: promote proliferation, migration and invasion, inhibit apoptosis	Inhibition of miR-7 → upregulation of RELA → promote tumor
		in vitro: promote proliferation, inhibit apoptosis	Unknown
hsa_circ_0001946	Promote tumor	In vitro: promote growth and inhibit apoptosis; in vivo: promote growth	Inhibition of miR-135a-5p → upregulation of SIRT1 → upregulation of β-catenin, c-myc and cyclin D1 → promote tumor
hsa_circ_0003645	Promote tumor	In vitro: promote growth, migration and invasion	Inhibition of miR-1179 → upregulation of TMEM14A → promote tumor
hsa_circ_0003998	Promote tumor	In vitro: promote proliferation and invasion	Inhibition of miR-326 → upregulation of Notch1 → promote tumor
hsa_circ_0004015	Promote tumor	In vitro: promote viability, proliferation and invasion, enhance resistance to gefitinib; in vivo: promote growth	Inhibition of miR-1183 → upregulation of PDPK1 → promote tumor
hsa_circ_0007534	Promote tumor	In vitro: promote proliferation, migration, invasion and epithelial-mesenchymal transition, inhibit apoptosis; in vivo: promote growth, epithelial-mesenchymal transition	Unknown
hsa_circ_000984	Promote tumor	In vitro: promote growth, migration, invasion and epithelial-mesenchymal transition, inhibit apoptosis	Upregulation of β-catenin, c-myc and cyclin D1 → promote tumor
hsa_circ_001569	Promote tumor	In vitro: promote proliferation	Upregulation of WNT1, β–catenin and TCF4 → promote tumor
hsa_circ_0016760	Promote tumor	In vitro: promote proliferation, migration and invasion, inhibit apoptosis; in vivo: promote growth	Inhibition of miR-1287 → upregulation of GAGE1 → promote tumor
hsa_circ_0020123	Promote tumor	In vitro and in vivo: promote proliferation, migration and invasion, inhibit apoptosis	Inhibition of miR-144 → upregulation of ZEB1 and EZH2 → promote tumor
		in vitro: promote growth, migration and invasion, inhibit apoptosis	Inhibition of miR-488-3p → upregulation of ADAM9 → promote tumor
hsa_circ_0020732	Promote tumor	In vitro: promote migration and invasion; in vivo: promote metastasis	inhibition of miR-665 → upregulation of ZEB1 → promote tumor
hsa_circ_0023404	Promote tumor	In vitro: promote growth, migration, invasion and epithelial-mesenchymal transition	Inhibition of miR-217 → upregulation of ZEB1 → promote tumor
hsa_circ_0025033	Promote tumor	In vitro: promote growth, migration and invasion, inhibit apoptosis	Inhibition of miR-1304-5p → upregulation of PPDPF and MACC1 → promote tumor
hsa_circ_0067934	Promote tumor	In vitro: promote proliferation	Unknown
		in vitro: promote proliferation, migration and invasion, epithelial-mesenchymal transition	upregulation of N-cadherin and vimentin, downregulation of E-cadherin → promote tumor
hsa_circ_0087862	Promote tumor	In vitro: promote growth, migration and invasion, inhibit apoptosis	Inhibition of miR-593-3p and miR-653-5p → upregulation of CCND2 and TIAM1 → promote tumor
hsa_circ_100833	Promote tumor	In vitro: promote proliferation and invasion	Inhibition of miR-498 → promote tumor
hsa_circ_100876	Promote tumor	Unknown	Unknown
hsa_circ_102231	Promote tumor	In vitro: promote proliferation and invasion	Unknown
hsa_circ_103809	Promote tumor	In vitro: promote proliferation and invasion; in vivo: promote growth	Inhibition of miR-4302 → upregulation of ZNF121 → upregulation of MYC → promote tumor
hsa_circ_103827	Promote tumor	Unknown	Unknown
hsa_circ_000122	Suppress tumor	Unknown	Unknown
hsa_circ_0001649	Suppress tumor	In vitro and in vivo: inhibit growth and metastasis	Inhibition of miR-331-3p and miR-338-5p → suppress tumor
hsa_circ_0002346	Suppress tumor	In vitro: inhibit migration, invasion and epithelial-mesenchymal transition; in vivo: inhibit metastasis	Inhibition of miR-93 and miR-182 → upregulation of LIFR → suppress tumor
hsa_circ_0006427	Suppress tumor	In vitro: inhibit proliferation, migration and invasion, epithelial-mesenchymal transition; in vivo: inhibit growth and epithelial-mesenchymal transition	Inhibition of miR-6783-3p → upregulation of DKK1, downregulation of β-catenin, c-myc and cyclin D1 → suppress tumor
hsa_circ_0007874	Suppress tumor	In vitro and in vivo: inhibit growth	Inhibition of miR-17 → upregulation of QKI-5 → downregulation of NICD, HES1 and Hey2 → suppress tumor
hsa_circ_0046264	Suppress tumor	In vitro: induce apoptosis, inhibit proliferation and invasion; in vivo: inhibit growth	Inhibition of miR-1245 → upregulation of BRCA2 → suppress tumor
hsa_circ_100395	Suppress tumor	In vitro: inhibit proliferation, migration and invasion, arrest cell-cycle progression; in vivo: inhibit growth	Inhibition of miR-1228 → upregulation of TCF21 → suppress tumor

AKT = protein kinase B, CCND2 = cyclin D2, CCNE1 = cyclin E1, circRNA = circular RNA, EGFR = epidermal growth factor receptor, EZH2 = enhancer of zeste homolog 2, Gal1 = galectin-1, LARP1 = La-related protein 1, LIFR = leukemia inhibitory factor receptor, MACC1 = metastasis-associated in colon cancer 1, NICD = Notch intracellular domain, PDPK1 = 3-phosphoinositide dependent protein kinase-1, PIK3CD = phosphoinositide 3-kinase catalytic subunit delta, PPDPF = pancreatic progenitor cell differentiation and proliferation factor, SIRT1 = sirtuin 1, TCF4 = transcription factor 4, TIAM1 = T-cell lymphoma invasion and metastasis 1, TMEM14A = transmembrane protein 14A, ZEB1 = zinc finger E-box binding homeobox 1.

Detailed molecular mechanisms of tumor promoting circRNAs in lung carcinogenesis are summarized in Table 7 and are discussed in the following 6 paragraphs.

Circ-BANP functions as a miR-503 sponge, and miR-503 targets 3′-untranslated region (UTR) of La-related protein 1 mRNA.^[39]^ La-related protein 1has been validated as an oncogene in NSCLC, and it stabilizes mammalian target of rapamycin (mTOR), sustaining mTOR signaling and promoting cancer cell growth.^[40,41]^ CircFGFR3 acts as a miR-22-3p sponge, and miR-22-3p targets galectin-1 mRNA.^[42]^ galectin-1mediates phosphorylation of protein kinase B (AKT) and extracellular signal-regulated kinase 1/2 by circFGFR3, which is required for tumor-promoting effects in NSCLC cell models 95D and A549. CircPRKCI is a sponge for both miR-545 and miR-589.^[43]^ Increase of circPRKCI expression blocks the inhibitory effect of these 2 miRNAs on tumorigenic transcription factor E2F7. Because E2F7 negatively regulates P21, cyclin D1, downstream of P21, is upregulated. Increase of cyclin D1 accelerates and disrupts cell cycle by promoting G1/S phase transition, thus inducing tumorigenesis.^[44,45]^ High level of E2F7 has also been observed in NSCLC patients with poor prognosis.^[46]^ Circ-PRMT5 acts as the sponge for miR-377, miR-382, and miR-498.^[47]^ All the 3 miRNAs bind with 3′-UTR of oncogene enhancer of zeste homolog 2 (EZH2) transcripts and inhibit EZH2 mRNA translation.^[47]^ EZH2 is a lysine methyltransferase and regulates chromatin function.^[48]^ It disturbs cellular metabolism and promotes tumor angiogenesis.^[49]^ Increase of EZH2 expression has been observed in NSCLC and is associated with decreased survival.^[50]^ CircPVT1 binds with miR-497 as its sponge.^[51]^ MiR-497 directly targets Bcl-2, which encodes a well-known antiapoptotic and oncogenic protein.^[52]^ Therefore, increase of cirPVT1 induces cancer cells to become apoptosis-resistant both in vitro and in vivo.

CiRS-7 targets miR-7. MiR-7 is a key tumor suppressor.^[53]^ Suppression of miR-7 promotes cell proliferation and inhibits apoptosis by increasing EGFR, cyclin E1 (CCNE1), and phosphoinositide 3-kinase catalytic subunit delta.^[54]^ EGFR overexpression is observed in 40% to 80% of patients with NSCLC.^[55]^ Activation of EGFR signaling increases expression of genes that regulate cell proliferation, invasion, migration, and angiogenesis.^[56]^ CCNE1 is a cell cycle regulator in G1/S transition, and its inhibition via miR-7 leads to cell cycle arrest in G1 phase.^[57]^ Overexpression of phosphoinositide 3-kinase catalytic subunit delta affects both PI3K/AKT pathway and RAS pathway, leading to increase of cell proliferation.^[58]^ Inhibition of miR-7 also results in increased viability, invasion, and migration of A549 and H1299 cells by upregulating RELA, a subunit of nuclear factor-kappa B (NF-κB).^[59]^ A meta-analysis exhibits higher NF-κB expression is associated with higher tumor stage, lymph node metastasis, and shorter OS of NSCLC patients.^[60]^ Mechanistically, NF-κB induces cyclins D and E, and suppresses checkpoint protein GADD45, thus disrupting cell cycle and promoting lung carcinogenesis.^[61]^ Moreover, NF-κB is involved in tumor resistance to chemotherapy and radiotherapy.^[62]^ The third study of ciRS-7 does not explore the mechanism.^[63]^ Hsa_circ_0001946 inhibits miR-135a-5p, resulting in upregulation of sirtuin 1.^[64]^ Sirtuin 1 deacetylates β-catenin and activates Wnt/β-catenin signaling pathway.^[65,66]^ Disruption of Wnt/β-catenin pathway promotes lung tumorigenesis and relates to drug resistance and poor prognosis.^[67,68]^

Hsa_circ_0003645 is a miR-1179 sponge while miR-1179 targets transmembrane protein 14A (TMEM14A).^[69]^ Therefore, upregulation of hsa_circ_0003645 correlates with upregulation of TMEM14A. TMEM14A is desregulated in multiple cancers.^[70]^ Knockdown of TMEM14A in ovarian cancer A2780 and HO-8910 cells downregulates TGF-β/Smad signaling, arrests cell cycle and suppresses cell proliferation, migration and invasion, suggesting the oncogenic role of TMEM14A.^[71]^ Hsa_circ_0003998 is a competing endogenous RNA for miRNA-326.^[72]^ Its increase leads to blocking of the inhibitory effect of miRNA-326 on Notch1. Notch1 has been associated with an increased chance of lymph node metastasis and decreased OS in NSCLC patients.^[73]^ Functionally, suppression of p53-mediated apoptosis by Notch1 is required for tumor initiation, and Notch1 promotes NSCLC cell survival via upregulation of insulin-like growth factor 1 receptor under hypoxia.^[74,75]^ Hsa_circ_0004015 is a sponge for miR-1183, while 3-phosphoinositide dependent protein kinase-1 is a target of miR-1183.^[76]^ 3-Phosphoinositide dependent protein kinase-1phosphorylates AKT and subsequently activates mTORC1.^[77]^ Activation of AKT/mTOR signaling is frequently observed and confers resistance to EGFR inhibitor in NSCLC.^[78]^ Hsa_circ_000984 upregulates β-catenin, c-myc, and cyclin D1.^[79]^ Amplification of c-myc is observed in human lung cancer cell lines and c-myc copy number gain is an independent factor predicting poor prognosis in lung adenocarcinoma.^[80,81]^ Cyclin D1 is a critical driver of malignant transformation in NSCLC.^[82]^ Its expression correlates with altered p53 expression, and higher cyclin D1 level promotes cancer cell proliferation.^[83]^

Knockdown of circ_001569 decreases oncogenic protein WNT1, β-catenin, and transcription factor 4 in A549 and H1299 cells.^[84]^ Wnt/TCF activation increases the risk of brain metastases and predicts shorter survival in patients with LUAD.^[85]^ HOXB9 and LEF1, which are downstream target genes of Wnt/TCF signaling, also mediate chemotactic invasion and colony outgrowth in H2030-BrM3 cell.^[86]^ Hsa_circ_0016760 directly sponges and suppresses miR-1287.^[87]^ This results in upregulation of GAGE1. GAGE1 is a member of cancer/testis antigens.^[88]^ Proteins in GAGE family are only expressed in cancer and germ cells, which makes them good candidates for immunotherapy. GAGE has also been shown to express in NSCLC tissues, and higher level indicates advanced clinical stages.^[89]^ Hsa_circ_0020123 inhibits miR-144.^[90]^ Inhibition of miR-144 promotes expression of zinc finger E-box-binding homeobox 1 (ZEB1), and ZEB1 promotes tumor invasion and migration by inducing epithelial mesenchymal transition.^[91]^ Another miR-144 target EZH2 is a histone methyltransferase. By epigenetic modification, EZH2 benefits cancer cell survival, induces epithelial mesenchymal transition, and confers drug resistance.^[92]^ Hsa_circ_0020123 is also a sponge for miR-488-3p, while miR-488-3p inhibits ADAM9 translation.^[93]^ Overexpression of ADAM9 stimulates expression of vascular endothelial growth factor A, increases angiogenesis, promotes vascular remodeling, and correlates with metastasis and poor prognosis in lung cancer.^[94,95]^

Hsa_circ_0020732 sponges miR-665, and inhibition of miR-665 results in upregulation of ZEB1.^[96]^ Increase of ZEB1 promotes lung cancer metastasis via inducing EMT.^[97]^ ZEB1 also mediates acquired resistance to EGFR-tyrosine kinase inhibitors in NSCLC.^[98]^ Furthermore, ZEB1 expression level is related to NSCLC clinical stage, tumor size, and patient survival.^[99]^ Hsa_circ_0023404 sponges miR-217, which is also predicted to target ZEB1.^[100]^ Therefore, upregulation of hsa_circ_0023404 results in inhibition of miR-217 and upregulation of ZEB1.^[100]^ Upregulation of hsa_circ_0025033 inhibits miR-1304-5p, which further results in upregulation of pancreatic progenitor cell differentiation and proliferation factor and metastasis-associated in colon cancer 1.^[101]^ pancreatic progenitor cell differentiation and proliferation factor is upregulated in liver cancer and correlates with cancer progression and lower survival.^[102]^ Higher metastasis-associated in colon cancer 1expression is associated with higher tumor grade, lymph node metastasis, and poorer disease-free survival in NSCLC.^[103,104]^ Knockdown of Hsa_circ_0067934 increases epithelial marker E-cadherin and decreases mesenchymal markers N-cadherin and vimentin.^[105]^ Therefore, hsa_circ_0067934 induces EMT to promote NSCLC metastasis. The second study of hsa_circ_0067934 does not include functional mechanism.^[106]^

Hsa_circ_0087862 sponges miR-593-3p and miR-653-5p.^[107]^ MiR-593-3p targets cyclin D2, and miR-653-5p targets T-cell lymphoma invasion and metastasis 1.^[107]^ Cyclin D2 plays an important role in cell cycle arrest and is involved in NSCLC oncogenesis.^[108]^ T-cell lymphoma invasion and metastasis 1stimulates EMT and angiogenesis in lung adenocarcinoma and its overexpression indicates poor prognosis.^[109]^ Hsa_circ_100833 serves as a miR-498 sponge.^[110]^ MiR-498 expression is decreased in NSCLC and correlated with sub-classified tumor histology and T stage.^[111]^ MiR-498 also inhibits proliferation of A549 or H661 cells.^[111]^ Hsa_circ_103809 is a sponge of miR-4302 targeting zinc finger transcription factor ZNF121.^[112]^ ZNF121 interacts with another transcription factor MYC, and their expressions positively correlate with each other.^[113]^ MYC is a classic oncoprotein and promotes metastasis of NSCLC.^[114]^ The mechanisms of hsa_circ_0007534, hsa_circ_100876, hsa_circ_102231, and hsa_circ_103827 remain to be explored.^[115–118]^

On the other hand, we also discuss the major mechanisms of tumor suppressing circRNAs in lung carcinogenesis in Table 7. Hsa_circ_0001649 is identified as a sponge for both miR-331-3p and miR-338-5p.^[119]^ Overexpression of miR-331-3p has been detected in asbestos-related lung cancer, indicating its oncogenic potential.^[120]^ Expression of miR-338-5p is positively correlated with advanced tumor stage and metastasis.^[121]^ Mimics of these 2 miRs also restore cancerous proliferation and invasion of A549 and H1299 cells. Hsa_circ_0002346 sponges miR-93 and miR-182, both of which target leukemia inhibitory factor receptor (LIFR).^[122]^ Therefore, downregulation of hsa_circ_0002346 decreases LIFR expression. LIFR inhibits tumor metastasis via the Hippo-YAP pathway, and this tumor suppressive role of LIFR has been observed in multiple cancer, including lung cancer.^[123,124]^ Hsa_circ_0006427 serves as a miR-6783-3p sponge.^[125]^ MiR-6783-3p targets a Wnt/β-catenin pathway inhibitor DKK1. Because Wnt signaling pathway impacts NSCLC tumorigenesis, prognosis and therapy resistance, inactivation of Wnt/β-catenin signaling by miR-6783-3p inhibition results in tumor suppression.^[126]^ Hsa_circ_0007874 functions as a miR-17 sponge.^[127]^ Inhibition of miR-17 results in upregulation of QKI-5, further resulting in downregulation of Notch intracellular domain and 2 downstream genes of Notch pathway, HES1 and Hey2.^[127]^ Notch signaling plays multiple roles in lung cancer tumorigenesis and is associated with survival.^[128]^ Thus, inhibition of Notch signaling might suppress lung cancer. Hsa_circ_0046264 is a sponge for miR-1245.^[129]^ Inhibition of miR-1245 upregulates its target BRCA2. BRCA2 is a DNA double-strand break repair gene and a tumor suppressor. Low expression of BRCA2 has been observed in LUAD.^[130]^ Hsa_circ_100395 functions as a sponge for miR-1228 targeting TCF21 in lung cancer.^[131]^ Decrease of TCF21 mRNA level is predictive of poor prognosis in patients with LUAD.^[132]^ TCF21 overexpression in H1299 cell has also been shown to suppress tumor growth in a mouse model.^[133]^ The mechanism of hsa_circ_000122 is unknown.^[118]^

Other people have also explored the role of circRNAs in lung cancer.^[30,134]^ In a previous review article, Yang listed the biological mechanisms of 24 circRNAs in lung cancer development.^[30]^ Among them, 6 were found to have diagnostic value for NSCLC, and only 9 had the potential to predict prognosis. Clinical significance of other listed circRNAs was not uncovered. Since then, studies in this field have been burgeoning, especially the research focusing on the prognostic value of circRNAs in lung cancer. Thus, we conducted this systematic review. Apart from summarizing lung cancer-associated circRNAs with prognostic values, we further summarized their clinicopathological significance, and found the 2 most striking clinicopathological characteristics were lymph node metastasis and TNM stage, confirming the major role of circRNAs in lung cancer is promoting tumor invasion and migration. This role has also been proposed by other researchers for other types of cancer such as colorectal and hepatocellular carcinomas.^[135,136]^

There are several limitations of this study. First, the population is confined to the Chinese as all the original studies included were conducted in hospitals in China by Chinese physicians. Precautions need to be taken when the results are applied to other ethnicities. Second, research of the role of circRNA in cancer is still in the early stage. So far, the biological mechanisms of those prognosis-predictive circRNAs are all based on the basic function of circRNAs as miRNA sponges. However, other mechanisms, including function of acting as protein sponges, decoys and scaffolds, regulation of parental gene transcription and modulation of mRNA alternative splicing and stability, are also involved in cancer development.^[18,137]^ Whether circRNAs with such biological roles are related to clinicopathological characteristics and prognosis of lung cancer remains to be explored.

## 5 Conclusion

In conclusion, this study emphasizes the clinicopathological significance of circRNAs in Chinese populations that changes of certain circRNA expression levels are associated with lung cancer progression and differentiation. Changes of those circRNA expression are also predictive of survival of lung cancer patients. Functionally, the majority of circRNAs are associated with lung cancer proliferation, metastasis, and invasion. The specific biological role of each circRNA is predominantly based on its function as the miRNA sponge and dependent on its interactive miRNAs and the following signaling pathways. Understanding the biological and clinical roles of circRNAs will lay the foundation and provide a novel aspect to screen potential targets for lung cancer treatment in the future.

## Author contributions

**Conceptualization:** Yuxuan Zheng, Jie Hu, Ran Hao, Yixin Qi.

**Data curation:** Yuxuan Zheng, Yishuai Li, Ran Hao.

**Formal analysis:** Yuxuan Zheng, Yishuai Li.

**Funding acquisition:** Yixin Qi.

**Investigation:** Yuxuan Zheng, Ran Hao.

**Project administration:** Jie Hu, Ran Hao, Yixin Qi.

**Resources:** Jie Hu.

**Software:** Jie Hu, Ran Hao.

**Supervision:** Jie Hu, Ran Hao, Yixin Qi.

**Writing – original draft:** Yuxuan Zheng.

**Writing – review & editing:** Yuxuan Zheng, Jie Hu, Yishuai Li, Ran Hao, Yixin Qi.
